# Resveratrol and Depression in Animal Models: A Systematic Review of the Biological Mechanisms

**DOI:** 10.3390/molecules23092197

**Published:** 2018-08-30

**Authors:** Alyssa Moore, Joshua Beidler, Mee Young Hong

**Affiliations:** School of Exercise and Nutritional Sciences, San Diego State University, San Diego, CA 92182, USA; alyssamoore004@gmail.com (A.M.); beidler@gmail.com (J.B.)

**Keywords:** resveratrol, depression, anxiety, stress, neurogenesis, BDNF, inflammation

## Abstract

Depression is currently treated by pharmacotherapies that can elicit debilitating side effects for patients. Novel treatment options with limited side effects are currently being researched. Resveratrol is a polyphenol and phytoalexin found in the skins of grapes, red wine, Japanese knotweed, and peanuts. It has been studied extensively for its antioxidant and anti-inflammatory properties. Resveratrol has also gained attention for its neuroprotective properties. The aim of the review was to examine the mechanisms by which resveratrol reduces depressive behaviors in animal models. In total, 22 studies met the established criteria for final review. Behavioral aspects of depression were investigated using validated measures such as the forced swimming test, tail suspension test, sucrose preference test, and open field test. While many physical measures were taken, three main biological mechanisms were explored: Regulation of the hypothalamic–pituitary–adrenal axis; decreased inflammation; and increased Brain-Derived Neurotrophic Factor and neurogenesis. Based on these findings, resveratrol may be deemed an effective treatment for depression in animal models at doses between 10–80 mg/kg/day, although higher doses had the most significant effects. Future studies should examine the effects of resveratrol on depression in humans to determine the eligibility of resveratrol as a natural antidepressant with less severe side effects.

## 1. Introduction

Depression, the most common mental disorder, affects over 300 million individuals worldwide [1]. Mental, neurological, and substance-use disorders make up 13% of the global burden of disease, and depression is recognized as the third leading contributor. Depression has negative implications for individuals and their families and is associated with increased risk of mortality, lower income, higher unemployment, chronic disease, and other mental health disorders [2]. Furthermore, depression often leads to suicide, with 800,000 individuals dying due to suicide per year [1]. Depression also creates a major economic burden, which was estimated at $210.5 billion in 2010 [3]. According to the Centers for Disease Control and Prevention (CDC), 11 percent of Americans over the age of 12 take antidepressant medication; from 2005 to 2008, antidepressant use increased 400% across all age groups [3,4].

While the pathogenesis of depression is known to include genetic, environmental, and psychosocial factors, the exact biological mechanisms remain to be elucidated [5]. Research on classical antidepressants reveals that multiple factors are involved and several brain regions are affected, helping to establish the mechanisms at play. These include dysregulation of the hypothalamic-pituitary-adrenal (HPA) axis, decreased neurogenesis, oxidative stress, and changes in serotonergic and adrenergic pathways [6].

Several antidepressant classes exist, including monoamine oxidase inhibitors (MAOIs), tricyclic antidepressants (TCAs), selective serotonin reuptake inhibitors (SSRIs), and serotonin and norepinephrine reuptake inhibitors (SNRIs) [7]. TCAs effectively inhibit the reuptake of serotonin, norepinephrine, and dopamine, but also affect other receptor systems and therefore have substantial adverse side effects. SSRIs reduce the reuptake of serotonin, and SNRIs reduce the reuptake of both serotonin and norepinephrine. SSRIs are now the most commonly prescribed antidepressants, as they have similar effectiveness as other medications while posing fewer severe side effects. Still, about half of patients experience a full remission, and the side effects of SSRIs, which include gastrointestinal issues, weight gain, sleep disturbances, and sexual dysfunction, can significantly impact quality of life [8]. Therefore, it is imperative to examine novel therapeutic agents with limited side effects for the treatment of depression.

There is growing evidence for the value of botanical compounds and other natural substances in the treatment of psychiatric disorders. In addition to resveratrol, plant components with possible antidepressant effects include anthocyanidins, catechins, and cocoa [9]. Resveratrol (3,5,4′-trihydroxystilbene) is a phytoalexin and polyphenol found predominantly in the skins of red grapes, red wine, Japanese knotweed, and some nuts [10]. It has been studied extensively for its antioxidant, anti-inflammatory, and anticarcinogenic properties [11,12]. Resveratrol has also been implicated as a neuroprotective agent with the ability to increase neurogenesis, most notably in reducing Alzheimer’s disease progression [13]. More recently, resveratrol has been examined as a potential aid to improve sleep quality [14], reduce fatigue [15], and ameliorate anxiety and depression [16]. While resveratrol has been shown to decrease depressive behaviors and biochemical markers associated with depression in animal models, few human studies exist to replicate these findings. The objective of the present review was to examine the effects of resveratrol on depressive behaviors in animal models through mechanisms that include regulation of the HPA axis, reduced inflammation and oxidative stress, increased neurogenesis, and increased monoamine production.

## 2. Methods

**Figure 1:**
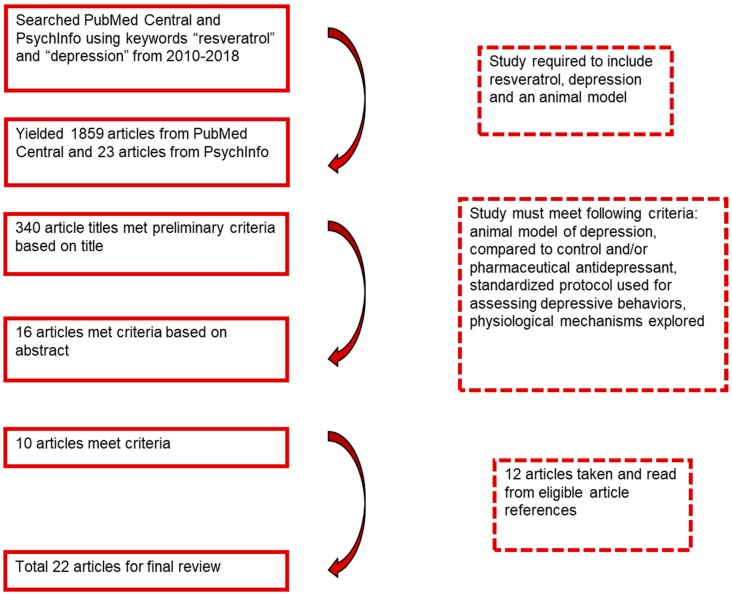
Process of article selection for final review.

Articles were searched for using the databases PubMed Central and PsycInfo with the latest search date of August 2018. The phrases “resveratrol” and “depression” were entered into the search engine with the limitations of the years 2010 to 2018. This yielded 1859 articles from PubMed Central and 23 articles from PsycInfo. The titles of the articles were reviewed to determine if the study involved resveratrol and depression using an animal model. Based on this, 340 articles were chosen, and their abstracts were reviewed to inquire if they included resveratrol related to behavioral symptoms of depression or physical parameters of depression. Sixteen articles were read in depth to determine if they (a) included an animal model of depression treated with resveratrol and compared to a control and/or pharmaceutical antidepressant; (b) examined depressive behaviors using standardized protocols; and (c) explored the physiological mechanisms. Ten articles were established as eligible based on these methods, while 12 articles were chosen from the references of others, yielding a total of 22 articles for final review (Figure 1). The results of each study were reported based on mean differences with an alpha level of 0.01 or 0.05 based on the parameters set by each study author. Some studies had a broader focus that did not fit within the context of this review, and thus only those results that corresponded to our specific objectives were cited (Table 1).

## 3. Results

### 3.1. Resveratrol′s Effect on Depressive Behaviors

Several different tests were utilized to investigate the effects of resveratrol on depressive behaviors, including the forced swimming test (FST), tail suspension test (TST), sucrose preference test, and open field test (OFT). The FST and TST assess helplessness or behavioral despair, with higher immobility time indicating greater depressive behaviors [35]. Decreased sucrose preference indicates anhedonia, a common symptom of depression defined as the loss of the ability to feel pleasure [18]. The OFT measures locomotor activity, with reduced locomotor activity indicating anxiety-like behaviors associated with depression [28]. Other studies used the OFT as a measure of resveratrol’s specificity, as psychostimulants similarly decrease immobility time in the FST and TST, but cause an increase in locomotor activity [7,35,36].

In animal models of depression, resveratrol increases sucrose consumption in a dose-dependent manner, demonstrating resveratrol’s ability to counteract the reduction in reward-seeking behavior that tends to occur with depression [16,18,21,22,26,28]. Animals also exhibit decreased immobility time in the FST and TST, with resveratrol treatment ranging from 15–80 mg/kg/day [5,6,16,18,21,22,24,26,28,32]. Moreover, resveratrol has been shown to reduce depressive behaviors in rodents to a similar degree as the antidepressants fluoxetine, desipramine, and ketamine [7,16,18,21,26,28,32]. Several studies reveal that resveratrol has no effect on locomotor activity in the OFT, indicating its specificity [6,7,22,32,33], while others show a reversal of decreased locomotor activity, demonstrating resveratrol’s ability to reduce anxiety-like behaviors associated with depression [16,28].

### 3.2. HPA Axis Regulation

The HPA axis is a major endocrine system in the body that regulates how individuals adapt and behave in the face of stress. When a stressor presents, the paraventricular nucleus (PVN) of the hypothalamus releases corticotropin-releasing hormone (CRH), which stimulates the release of adrenocorticotropic hormone (ACTH) from the anterior pituitary. ACTH enters the bloodstream and causes the release of glucocorticoids (cortisol in humans and corticosterone in animals) from the adrenal glands [37].

Studies show that serum and plasma cortisol is higher in individuals suffering from major depression [38,39,40]. Resveratrol attenuates this increase in serum and plasma corticosterone in several animal models of depression [16,18,21,26,32]. In one study, mice receiving corticosterone had significantly higher serum corticosterone levels than control animals, but resveratrol (80 mg/kg/day) reversed this effect similarly to fluoxetine [18]. Wang and colleagues also found that mice induced with stress via the FST and TST had lower serum corticosterone after 21 days of either resveratrol or fluoxetine treatment than vehicle mice [32].

In a rat model of subclinical hypothyroidism-induced depression, resveratrol not only reduced plasma corticosterone levels and depressive behaviors, but also hypothalamic CRH mRNA expression and levels of thyrotropin-releasing hormone (TRH) and thyroid stimulating hormone (TSH) [16]. These results support the direct effect of resveratrol on hypothalamic activity. In contrast, others have recognized a decrease in serum corticosterone but no change in CRH mRNA expression with resveratrol treatment [21]. Together, these results illustrate the beneficial effects of resveratrol on regulating the HPA axis in animal models of depression.

### 3.3. Decreased Inflammation

Chronic inflammation has been associated with both psychological stress and the pathology of depression [40]. Medically ill patients with increased inflammatory cytokines have higher rates of depression, and even in the absence of medical illness, individuals with depression exhibit increased pro-inflammatory cytokines [41]. Pathogens or stress trigger a signaling cascade which activates transcription factors such as nuclear factor-κB (NF-κB), which in turn increase the expression of pro-inflammatory genes [40].

Pro-inflammatory cytokines, including interleukin-1 (IL-1), interleukin-6 (IL-6), and tumor necrosis factor-α (TNF-α), play a key role in the immune and inflammatory response. While pro-inflammatory cytokines work to mitigate infection in the short term, continuous inflammation resulting from prolonged activation can lead to poor health outcomes and depression. In fact, one way that pro-inflammatory factors attempt to protect the organism is by promoting social withdrawal, a behavior displayed by depressed patients [40]. Resveratrol is able to downregulate NF-κB expression in the hippocampus [5,21] and prefrontal cortex [22] in mice treated with lipopolysaccharide (LPS). In addition, resveratrol supplementation reduces levels of pro-inflammatory cytokines (IL-1β, IL-2, IL-4, IL-6, TNF-α) in animal models of depression [20,22].

Resveratrol is able to reduce microglial activation and Iba1 labeling of microglia in the hippocampus, establishing its anti-inflammatory effect [5,24]. Microglia are immune cells that reside in the brain. Overactivation of microglia occurs when neurons are damaged and is associated with several neurodegenerative disorders including depression [42].

### 3.4. Decreased Oxidative Stress

Depression is characterized by increased oxidative stress, as indicated by higher levels of malondialdehyde (MDA) and 8-hydroxy-2-deoxyguanosine (8-OHdG). Some studies have also shown decreased antioxidant status, although these results are less consistent [43]. Although resveratrol’s direct antioxidant effects are modest, this polyphenol significantly reduces oxidative stress through its effects on gene expression, which include upregulation of antioxidant enzyme production and downregulation of reactive oxygen species (ROS) production [44]. Two studies in male rats exposed to chronic unpredictable mild stress (CUMS) showed reductions in oxidative stress following resveratrol treatment, which also reduced depression-like symptoms. Serum MDA was significantly reduced by 15 mg/kg/day of resveratrol, while a higher dosage (80 mg/kg/day) reduced MDA and increased superoxide dismutase (SOD) in the prefrontal cortex and hippocampus [21,28].

### 3.5. Decreased Amyloid Beta Cytotoxicity

Amyloid beta (Aβ), the primary component of plaques found in the brains of Alzheimer’s disease patients, may also promote depression by impairing function of the serotonin neurotransmitter system [45]. In rat and human cell cultures, resveratrol has demonstrated the ability to reduce Aβ levels and to attenuate Aβ-induced cytotoxicity and cell death [46,47,48,49]. Cells exposed to Aβ exhibited increased NF-κB activity, but pretreatment with resveratrol inhibited this effect [48]. These results suggest that resveratrol’s antidepressant effects may occur partly through a reduction in neural Aβ levels.

### 3.6. Increased Neurogenesis

#### 3.6.1. Brain-Derived Neurotrophic Factor (BDNF)

Brain-Derived Neurotrophic Factor (BDNF) is imperative for neuroprotection due to its roles in neuronal plasticity and survival, synaptic transmission, and neurotransmitter synthesis [50]. Chronic stress and glucocorticoids decrease BDNF [51,52], while it is believed that antidepressants exert their effects in part by increasing BDNF levels [53]. In rodents, resveratrol increases BDNF levels in various brain regions, including the hippocampus [6,18,22,26,32], prefrontal cortex [6,26,32], and amygdala [26]. Notably, when corticosterone was administered to mice, levels of BDNF decreased in the hippocampus, but resveratrol attenuated this effect similarly to fluoxetine [18]. Hurley and colleagues showed that BDNF was increased in the hippocampus by both acute resveratrol treatment, and chronic administration over a seven-day period [6].

#### 3.6.2. cAMP Response Element Binding Protein

Cyclic AMP response element-binding protein (CREB) is a transcription factor associated with depression [54]. Low levels of CREB activity are implicated in depression [54], while chronic administration of antidepressants increases CREB mRNA expression in various brain regions of rodents [55,56]. Phosphorylated CREB (pCREB) binds to a receptor on the promoter region of the BDNF gene, enhancing its transcription [22,57]. Resveratrol increased pCREB and BDNF in the hippocampus, prefrontal cortex, and amygdala of mice exposed to LPS [22] and rats exposed to CUMS [26]. Thus, resveratrol may act similarly to classical antidepressants by upregulating CREB activity, thereby increasing BDNF expression.

#### 3.6.3. Extracellular Regulated Kinase Pathway

Extracellular regulated kinase (ERK) signaling is involved in the survival and resilience of neurons [24]. In both humans and rodents, studies show that defects in the ERK signaling cascade are associated with depression [58,59]. In rats exposed to CUMS, antidepressants increase phosphorylation of ERK (pERK) [60] and hippocampal BDNF expression [61]. Resveratrol has also been shown to increase pERK in both the hippocampus [26,32,33] and prefrontal cortex [32,33] of rodents. Studies demonstrate that BDNF exerts antidepressant activity by upregulating the ERK pathway. Therefore, resveratrol may alleviate depressive symptoms both by increasing BDNF and by upregulating the ERK signaling cascade [26,32,33].

#### 3.6.4. Neural Stem Cells

Neural stem cells reside in the subgranular zone of the dentate gyrus. Radial glia-like (RGL) cells have a bipolar structure and function as the main progenitor cells for adult hippocampal neurogenesis [5]. In mice administered LPS, resveratrol increased the number of RGL cells by increasing symmetric division [5]. Not only did resveratrol treatment increase the number of newly born cells and neurons in the hippocampus, these neurons lasted for at least two weeks, demonstrating the long-term effects of resveratrol treatment [5,24].

#### 3.6.5. Mammalian Target of Rapamycin (mTOR) Pathway

Mammalian target of rapamycin (mTOR) is a serine/threonine protein kinase involved in cell proliferation, survival, and protein synthesis [62]. It has been elucidated that phosphorylation of the mTOR signaling pathway is severely decreased in patients with major depression, while ketamine, an *N*-methyl-d-aspartate (NMDA) antagonist, has been shown to increase mTOR activity and relieve depression [63]. Following phosphorylation, Akt/protein kinase B (Akt) activates the mTOR signaling pathway [28]. Resveratrol is comparable to ketamine in reducing depressive symptoms and increasing Akt and mTOR levels in the hippocampus and prefrontal cortex of rats [28]. Therefore, resveratrol may ameliorate depressive symptoms via phosphorylation of Akt and subsequent activation of the mTOR pathway.

#### 3.6.6. Canonical Pathway of Wnt-β-Catenin

Wnt-β-Catenin signaling plays a significant role in regulating hippocampal neurogenesis [64,65]. Dysregulation of this pathway is associated with several neuropsychiatric disorders including depression [65,66]. In addition, GSK-3β, a kinase involved in Wnt signaling, is used as a marker in neuropsychiatric disorders and is targeted by mood-stabilizing drugs such as lithium for bipolar disorder [65,67].

Rats with subclinical hypothyroidism-associated depression exhibited increased levels of GSK-3β and phosphorylated β-catenin, while c-myc and cyclin D1 were decreased. Resveratrol attenuated these effects by decreasing GSK-3β and phosphorylated β-catenin while increasing expression of the Wnt target genes c-myc and cyclin D1. These effects were accompanied by increased sucrose preference and decreased immobility time in the FST, indicating a reduction in behavioral symptoms of depression [16].

### 3.7. Serotonin, Norepinephrine, & Dopamine

Current pharmacotherapies for depression act on monoamine neurotransmitters, including serotonin and norepinephrine. SSRIs and SNRIs prevent reuptake of serotonin or norepinephrine, while MAOIs inhibit monoamine oxidase (MAO), the enzyme responsible for degrading serotonin and norepinephrine [68]. Dopamine, which is responsible for reward and motivation, has also been implicated in depression, as modulating dopamine-producing neurons can either induce or mitigate depressive behaviors such as anhedonia [69]. In rodents, resveratrol has been shown to increase serotonin in the frontal cortex, hippocampus, and striatum; norepinephrine in the frontal cortex and hippocampus; and dopamine in the frontal cortex and striatum [7,70]. In mice, resveratrol also decreased levels of the enzymes MAO A and B, which reduce neurotransmitter levels through oxidative deamination [7,23]. Treatment with resveratrol decreased depressive behaviors—however, the effects of resveratrol diminished in the absence of serotonin. Taken together, these results demonstrate that resveratrol may reduce depressive behaviors in mice via the serotonergic and noradrenergic systems [7].

**Figure 2:**
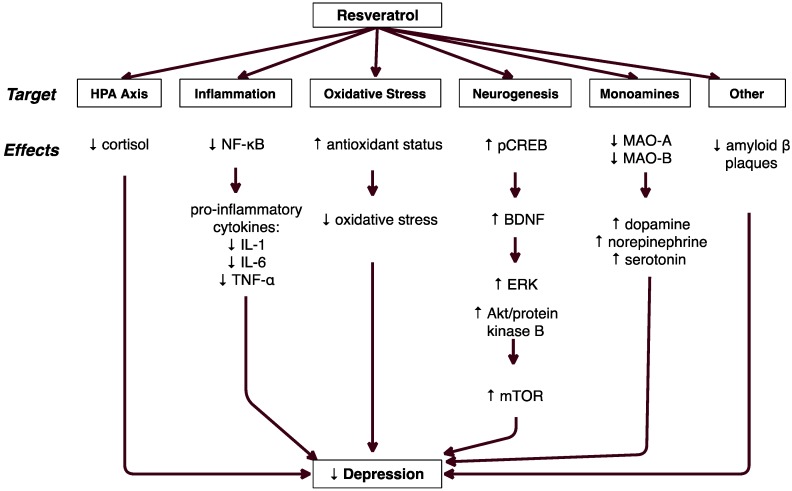
Proposed Mechanisms of Antidepressant Effects of Resveratrol.

Figure 2 shows the major proposed mechanisms by which resveratrol exerts antidepressant effects. These mechanisms include modulation of the hypothalamic-pituitary-adrenal (HPA) axis, reduction of inflammation and oxidative stress, increased neurogenesis, altered monoamine levels, and reduction in amyloid beta plaques.

## 4. Discussion

Based on the current literature, resveratrol is a promising novel therapy for the treatment of depression. This natural polyphenol decreases behavioral symptoms of depression and improves biological parameters associated with depression in animal models [5,6,7,16,18,20,21,22,24,26,28,32,33]. The effects of resveratrol treatment are similar to those of classical antidepressants [7,16,18,19,21,26,32], validating its potential use as a therapeutic agent. For many, the side effects of antidepressants are deleterious [8]. This has created an urgent need for new treatments that can improve the symptoms of depression without compromising wellbeing.

Importantly, resveratrol reduced depressive symptoms in all studies reviewed regardless of the type of stress induced. Models used in the studies included corticosterone injection [18], social stress exposure [20], CUMS [21,26,28], LPS injection [5,22], subclinical hypothyroidism [16], chronic restraint stress (CRS) [33], and stress induced by tests of behavioral despair [6,7,24,32]. The causes of depression are multifactorial, and it is evident that many brain regions and neural pathways are involved [6]. Resveratrol was found to decrease depressive symptoms in rodents by regulating the HPA axis, decreasing inflammation, and increasing neurogenesis.

The HPA axis is a hormonal stress response system. In the healthy individual, the HPA axis is regulated by negative feedback mechanisms that prevent cortisol overproduction [28]. In depressed individuals, the HPA axis is dysregulated, causing either chronically high cortisol levels or a flattened cortisol curve referred to as the diurnal slope [38,39,40,71]. Cortisol must effectively bind to its glucocorticoid receptor on the PVN and the anterior pituitary for inhibition to occur. It is speculated that improper binding of cortisol to its receptor during negative feedback causes excessive production of cortisol or an abnormal cortisol curve [37,72,73].

In one study, healthy premenopausal women or those diagnosed with major depression were administered dexamethasone, which binds to the glucocorticoid receptor and suppresses cortisol production. Although cortisol production was reduced in all participants, those diagnosed with major depression showed less suppression than non-depressed controls. In addition, depressed subjects had flatter diurnal cortisol curves than non-depressed subjects [72]. These results illustrate that HPA axis dysfunction is a strong indicator of depression.

As demonstrated by the present review, resveratrol effectively reduced corticosterone in several animal models of depression [16,18,21,26,32]. These results coincide with studies of classical antidepressants that exhibit decreased stress response and corticosterone levels in aquatic animals exposed to fluoxetine and diazepam [74]. Resveratrol decreased corticosterone levels similarly to SSRIs and simultaneously improved depressive behaviors. Thus, resveratrol may be considered a regulator of the HPA axis in its ability to reduce corticosterone in animal models of depression.

Much research has focused upon the association between inflammation and depression, and results show that regardless of a comorbid medical illness, inflammation is often present in depressed patients 41]. While it is unclear whether inflammation causes depression or the reverse, it is well known that pro-inflammatory cytokines have effects that could contribute to depressive symptoms [75]. For example, research shows that pro-inflammatory cytokines activate the HPA axis by stimulating CRH release by the hypothalamus and ACTH release by the pituitary, thereby increasing cortisol production [76]. Pro-inflammatory cytokines can also decrease serotonin synthesis in the brain and play a role in neuronal development and plasticity. Chronic stress activates microglia, which secrete pro-inflammatory cytokines that create inflammation and inhibit neurogenesis [75]. Depressed individuals exhibit upregulation of NF-κB, which is also involved in the release of pro-inflammatory cytokines. In vitro, resveratrol inhibited the activation of NF-κB by IL-1β [5,77]. The fact that resveratrol reduced both NF-κB expression and pro-inflammatory cytokines in animal models of depression suggests that resveratrol may attenuate the effects of inflammation on depression by downregulating the NF-κB signaling pathway.

Growing evidence suggests that oxidative stress is involved in the pathogenesis of depression. A recent meta-analysis of 23 observational studies found a significant association between depression and oxidative stress, and post-mortem studies have found indications of oxidative stress in the prefrontal cortex of patients with major depression [78,79]. MDA levels are positively associated with depression, particularly in patients who experience recurring episodes [34]. Another oxidative stress biomarker, 8-OHdG, is typically increased in depressed patients compared to healthy controls and correlates with the severity of depression [43]. Some studies show decreased levels of the SOD and glutathione, a finding consistent with the hypothesis that excessive ROS have overwhelmed the body’s antioxidant defenses. However, other studies have shown a positive relationship between antioxidant levels and depressive symptoms, and more research is needed to elucidate this relationship.

In addition to damaging cells, ROS can activate stress-responsive kinases including ERK, Jun N-terminal kinase (JNK), and p38, which in turn increase inflammation by activating NF-κB [80]. Resveratrol exerts antioxidant effects by scavenging free radicals, and through its effects on redox-related gene expression [44]. Although few of the studies in this review examined oxidative stress and antioxidant markers, two studies in CUMS-exposed male rats did show reductions in MDA following resveratrol treatment. In a study by Ge et al., 15 mg/kg/day of resveratrol significantly decreased MDA in serum [21]. Subsequently, Liu et al. found that 80 mg/kg/day of resveratrol reduced MDA and increased SOD in the prefrontal cortex and hippocampus [28]. Both studies also showed that resveratrol reduced depression-like symptoms, as indicated by sucrose preference, FST, and TST.

Depression affects up to 90% of Alzheimer’s disease (AD) patients and is considered a major risk factor for the disease [81]. Depression frequently precedes cognitive impairment in AD, possibly pointing to a common pathology underlying these psychiatric disorders [45]. One of the characteristic features of AD is the accumulation of plaques, which are composed primarily of the peptide amyloid beta (Aβ). Rather than simply being a marker of disease activity, Aβ appears to contribute directly to disease progression. For example, injected Aβ has been shown to impair memory and induce depressive-like behavior in mice [82]. Aβ appears to affect several of the mechanisms explored in this review. Like depression, AD is characterized by chronic inflammation and increased blood levels of proinflammatory cytokines [41,83]. In mice, intracerebroventricular infusion of Aβ was shown to increase mRNA expression and hippocampal levels of TNF-α [81]. Higher levels of TNF-α are associated with increased depressive-like behavior, as indicated by FST and SPT. However, when the mice were administered infliximab, an anti-TNF-α antibody, depression symptoms were reduced [81].

Increased production of proinflammatory cytokines is associated with altered serotonin (5-HT) metabolism. In mice, Aβ significantly decreased brain 5-HT levels, but pretreatment with 5-HT prevented Aβ-induced microglial activation and TNF-α production [81]. However, mice deficient in toll-like receptor 4 (TLR4^−/−^) did not exhibit reduced 5-HT levels or increased depressive-like behavior. TLR4 levels were also elevated in AD patients and transgenic mouse models of AD. These results indicate that TLR4 may play a key role in modulating the depression-promoting effects of Aβ in mice, possibly by upregulating NF-κB, which increases production of inflammatory cytokines.

Another link between AD and depression is increased oxidative stress caused by elevated levels of ROS [48]. Aβ increases oxidative stress, and has been shown to induce apoptosis by generating hydrogen peroxide [48]. Aβ has also been reported to modulate the HPA axis. In a rat model of AD, Aβ increased norepinephrine in the prefrontal cortex and hippocampus while decreasing production in the amygdala [84]. Interestingly, corticosterone is typically elevated in animal models of depression; however, plasma corticosterone was decreased in Aβ-treated rats [84]. Resveratrol has been shown to significantly reduce secreted and intracellular levels of amyloid beta [46,47,48,49]. In addition, resveratrol attenuated Aβ-induced cytotoxicity and apoptosis [46,47,48]. These findings suggest that resveratrol may reduce the neurotoxicity of Aβ through several mechanisms, thus contributing to the antidepressant effects of this polyphenol.

As reported, resveratrol increased BDNF expression and decreased behavioral symptoms in several animal models of depression [6,18,22,32,33]. Previous research contends that levels of BDNF are lower in depressed individuals and that antidepressants raise levels of BDNF. There is strong evidence supporting the existence of a relationship between depression and BDNF expression, but exactly how antidepressant medications and resveratrol are able to manipulate this relationship is still being researched. It has been postulated that low levels of BDNF are partly caused by high glucocorticoid production. It is worth noting that some studies demonstrated a decrease in serum corticosterone alongside increased BDNF following resveratrol treatment [18,26,32].

Additionally, it is speculated that BDNF is regulated by both CREB expression and the ERK signaling pathway [85]. Based on the evidence that low levels of phosphorylated CREB (pCREB) are implicated in depression, and that this protein enhances BDNF transcription, it is reasonable to consider that increasing pCREB would decrease depressive symptoms. As discussed, resveratrol upregulated pCREB and BDNF while decreasing depressive behaviors, so it is conceivable that resveratrol exerts its effects via this mechanism [22,57]. However, another study found that resveratrol lowered pCREB and BDNF and subsequently reduced the proliferation of neural progenitor cells in the hippocampus of healthy mice, so this mechanism remains unclear [86]. Likewise, deficiencies in ERK signaling are associated with depression, while antidepressants have been shown to upregulate both pERK and BDNF [58,60]. Since resveratrol had comparable effects to standard antidepressants in regulating ERK signaling, increasing BDNF, and decreasing depressive behaviors, we can conceptualize that this may be another method through which resveratrol alleviates depressive behaviors in animal models.

While the majority of studies focused upon resveratrol’s effects on the HPA axis, inflammation, and BDNF expression, a few studies explored different methods by which resveratrol may reduce depressive symptoms in animals. These included resveratrol’s effects on neural stem cells [5,24], the Wnt-β-catenin pathway [16], mTOR signaling [28], and monoamine synthesis [7].

Studies show that reduced neurogenesis is associated with mental illness and stress, and that inflammation inhibits neurogenesis. Antidepressants increase the proliferation, maturation, and survival of neurons in the hippocampus [87]. In congruence with these findings, resveratrol increased the symmetric division of RGLs, which contributes to neuronal proliferation [5]. Additionally, depression is associated with abnormalities in Wnt signaling, an essential pathway for neurogenesis [65]. Resveratrol increased neurogenesis by upregulating the Wnt signaling pathway. Resveratrol also increased mTOR signaling, which similarly promotes increased neurogenesis [62].

Resveratrol increased levels of 5-HT, norepinephrine, and dopamine in various brain regions associated with depression, which coincides with the monoamine hypothesis [88]. Depression is associated with low levels of dopamine, and irregularities in dopaminergic neurons that play a large role in reward and motivation may be responsible for the anhedonia exhibited by depressed patients [89]. Based on the large body of research that has implicated these monoamines in depression, resveratrol may decrease depressive behaviors in animals by increasing neurotransmitter levels.

In 12 animal studies, resveratrol was compared to an antidepressant drug as an active control (Table 2). Nine studies compared resveratrol or a combination of resveratrol and piperine to the SSRI fluoxetine. The TCA imipramine was employed in four studies [7,23,29,30] and desipramine in two studies [26,29]. Other drugs administered included the citalopram (SSRI) [29], moclobemide (MAOI) [7,82], nisoxetine (norepinephrine reuptake inhibitor) [29], buproprion and nomifensine (norepinephrine-dopamine reuptake inhibitor) [29], and ketamine (NMDA receptor antagonist) [28]. All studies administered only a single dosage of the pharmaceutical agent, but several dosages of resveratrol were typically given in order to evaluate dose-ranging effects. In general, resveratrol and the drugs were similarly effective with regard to depression-like behavior as indicated by sucrose preference test, FST, and TST. Resveratrol also had similar biochemical effects as the antidepressants, such as lower corticosterone [7,18,21,25,31,32] and higher 5-HT levels [17,23]. The majority of studies showed that resveratrol was most effective at 80 mg/kg/day. The Human Equivalent Dose (HED) of 80 mg/kg/day in rats is 12.9 mg/kg/day. Therefore, a 60 kg individual would require 775 mg of resveratrol per day to achieve similar results [90]. Based on these calculations, an effective dose of resveratrol would be more plausibly obtained through supplementation than through food or beverages.

There are limitations of this review that make it difficult to accurately compare results. Firstly, the different methods used to induce depression or stress in animal models may affect the outcomes. Secondly, some studies took physical measurements directly after injection of resveratrol, while others waited for a longer period. Thirdly, some studies examined the acute effects of resveratrol, while others looked at chronic effects. However, most studies did conduct the trials over a period of at least three weeks. Fourthly, the dose of resveratrol ranged from 10 mg/kg/day to 80 mg/kg/day. Although this variation in dosage permitted researchers to evaluate dose-dependent effects, it made direct comparison of the results more difficult. Fifth, there are not yet enough published studies on the subject to determine the overlying mechanisms with a high degree of certainty. Sixth, there is a lack of studies in female subjects. Although Wang et al. found that resveratrol reduced depression-like symptoms in female mice [34], all other animal studies that met our inclusion criteria were conducted in male subjects. The reliance on male subjects reflects a broader tendency within psychiatric research, as most animal models of depression were developed using male rodents, and only later applied to females. The absence of female subjects is a cause for concern, as sex differences exist regarding both depression and the response to antidepressant treatment. These differences occur not only in humans, where they may largely be explained by social and cultural factors, but also in animal models, where they suggest the role of innate neurobiological factors [91]. Male and female rats respond differently to widely-used animal models of depression, including chronic unpredictable mild stress (CUMS), forced swim test, and lipopolysaccharide (LPS) exposure [91]. Because of sex-linked differences, the results of the published studies may not be applicable to females. Future studies of resveratrol and depression should attempt to determine whether existing results can be replicated in female animals.

Animal models of depression are at best imperfect analogues of clinical depression, and therefore caution must be exercised when extrapolating the results of animal studies to humans. Wine shows more positive effects on mood than other alcoholic beverages—however, the large number of components in wine makes it difficult to determine whether resveratrol was responsible for the beneficial effects [92]. Few studies have specifically examined the effects of resveratrol on depression in humans, although several studies have measured mood following resveratrol treatment [93,94,95,96,97]. Two of those studies found no significant differences in any measure of mood [17,96,97], and one found reduced anxiety following resveratrol treatment, but no changes in depression or other mood components [94]. The lack of significance in the latter study may have resulted from the resveratrol dosage, which was lower in proportion to body weight than the dosages used in most animal studies. Unfortunately, these studies have limited relevance to this review because at baseline the subjects were healthy adults who were not experiencing depressive symptoms. The trial by Davinelli et al. was conducted in postmenopausal women [93]. At baseline, depression was reported by many subjects, but not all, as depression was only one of several symptoms used as inclusion criteria. Compared with the placebo, resveratrol significantly reduced depressive symptoms as measured by the Hamilton Rating Scale for Depression (HAM-D). In patients with Minimal Hepatic Encephalopathy (MHE), resveratrol treatment was also associated with decreased depression as measured by the Beck Depression Inventory [93]. In light of this limited evidence, more controlled clinical studies are needed to elucidate resveratrol’s antidepressant effects in humans.

In conclusion, resveratrol exhibited positive effects in animal models of depression comparable to the effects of several pharmaceutical antidepressants. Although these findings cannot be generalized to humans, results in animal models delineate the potential for resveratrol to serve as a natural antidepressant. Resveratrol’s excellent safety profile and limited side effects make it an appealing option for depression patients, either as an alternative or adjuvant to conventional therapies. The studies discussed in this review focused on various biological mechanisms, but it is likely that these mechanisms work in concert with one another. Because resveratrol affected many brain regions and neural pathways, this polyphenol may be beneficial in many cases of depression, even if the underlying causes are heterogeneous. Based on the present review, resveratrol merits further investigation as a possible therapeutic agent for the treatment of depression.

## Figures and Tables

**Table 1:** Effects of Resveratrol on Behavioral and Clinical Outcomes of Depression.

Author, Year	Animal Model	Intervention	Dosage/Route	Behavioral Outcomes w/RSV Txt	Clinical Outcomes w/RSV Txt
Ahmed and colleagues, 2014 [17]	reserpine-injected adult male Wistar rats	vehicle (saline + DMSO) reserpine reserpine + reserpine + RSV reserpine + resperpine + FLX × 3 days	RSV: 15, 30, 60 FLX: 24	↑ ambulation in OFT ^a,a (30/60)^ ↓ latency in OOFT ^a,a,a (15/30/60)^ FST ↓ immobility time in FST ^a,a,a (15/30/60)^	↑ NT levels: brain 5-HT ^a,a,a (15/30/60)^, dopamine ^a,a,a (15/30/60)^, and Norepinephrine ^a (60)^
Ali and colleagues, 2015 [18]	CORT-injected male Swiss albino mice	CORT (con) CORT + RSV CORT + FLX Vehicle (con) 21 days	CORT: 40, s.c. RSV: 80, oral FLX: 15, oral Vehicle, 0, oral	↑ sucrose preference ^c^ ↓ immobility time in FST ^b^ ↓ immobility time in TST ^a^	↓ serum CORT ^b^ ↑ hippocampal BDNF ^a^
Chen and colleagues, 2017 [19]	LPS-injected male ICR mice	vehicle (saline) LPS LPS + RSV tests after 24 h	RSV: 0.3, i.c.v.	↑ sucrose preference ^a^ ↓ immobility time in FST ^a^	↓ hippocampal superoxide ^a^ ↓ hippocampal apoptosis ^a^ ↑ hippocampal ATP production ^a^ ↑ hippocampal mitochondrial membrane potential ^a^
Finnel and colleagues, 2017 [20]	social stress-exposed male Sprague-Dawley rats & Long-Evans retired breeders	RSV + social stress Vehicle + social stress 5 days social stress, Txt 7 days pre-social stress & during	RSV: 10, 30, i.p. Vehicle: 0, i.p.	↑ sucrose preference ^c (30)^	↓ TNF-α ^c (10/30)^ ↓ IL-1β ^a,b (10/30)^ ↓ IL-6 ^c (10/30)^ ↓ IL-2 ^c (30)^↓ IL-4 ^c (30)^
Ge and colleagues, 2013 [21]	CUMS-exposed male Sprague-Dawley rats	CUMS + Vehicle CUMS + RSV CUMS + FLX CUMS 21 days, Txt days 14–21	RSV: 15, i.p. FLX: 2, i.p.	↑ sucrose preference ^a^ ↓ immobility time in FST ^c^ ↓ immobility time in TST ^c^	↓ serum CORT ^c^ ↓ serum MDA ^c^ ~ serum CRH ^d^
Ge and colleagues, 2015 [22]	LPS-injected adult male Kunming mice	Vehicle (saline) Vehicle + LPS RSV + Vehicle RSV + LPS	RSV: 80, i.p. LPS: 0.83, i.p.	↓ immobility time in FST ^b^ ↑ swimming time in FST ^b^ ↓ immobility time in TST ^a^ ↑ sucrose preference ^a^ ~ locomotor activity ^d^	↓ hippocampal & PFC IL-1β ^a^ ↓ hippocampal TNF-α ^a^ ↓ PFC TNF-α ^b^ ↓ hippocampal & PFC pNF-κB p65 ^a^ ↑ PFC pCREB ^a^ ↑ hippocampal BDNF ^a^
Ge and colleagues, 2016 [16]	SCH male Sprague-Dawley rats	SCH + Vehicle SCH + RSV SCH + LT4 Txt post SCH for 16 days	RSV: 15, i.g. LT4: 60 *, i.g.	↑ sucrose preference ^b^ ↑ locomotor activity ^a^ ↓ immobility time in FST ^b^ ↓ immobility time in TST ^b^	↓ adrenal gland wt to body wt ratio ^b^ ↓ plasma CORT ^b^ ↓ CRH mRNA expresión ^b^ ↓ GSK-3β ^b^ ↑ pGSK-3β ^b^ ↑ pGSK-3β/ GSK-3β ratio ^b^ ↑ β-catenin ^b^ ↓ p β-catenin ^b^ ↓ p β-catenin/ β-catenin ratio ^b^ ↑ cyclin D1 & c-myc ^b^ ↓ TSH ^a^ ↓ TRH mRNA expression ^b^
Huang and colleagues, 2013 [23]	male ICR mice	RSV FLX RSV + piperine	RSV: 1.25, 2.5, 10, 20, 40, 160, oral FLX: 10 i.p. piperine: 2.5 i.p.	↓ immobility time in FST ^a,a,a,a(2.5/10/40/160)^ ↓ immobility time in TST ^a,a,a,a(2.5/10/40/160)^ ~ locomotor activity^d^ ↓ reserpine-induced hypothermia ^a,a,a,a,a(1.25/2.5/5/10/20)^ and ptosis ^a (20)^	↑ 5-HT ^a,b (10/20)^, norepinephrine ^a (20)^, dopamine ^a (20)^ in FC ↓ 5-HIAA/5-HT in FC ^b (20)^ ↓ MAO-A in FC ^a,b,b (5/10/20)^ and hippocampus ^a,b (10/20)^ ↓ MAO-B in FC ^a (20)^
Hurley and colleagues, 2014 [6]	Adult male Wistar kyoto rats	RSV v Vehicle Txt 20 min post-injection (acute) & 18–20 h post-injection (chronic) × 7 days	RSV: 0 (saline), 10, 40, i.p.	↓ immobility time in FST ^a,c (acute, 10/40)^ ↓ immobility time in FST ^a,c (chronic, 10/40)^ ~ sucrose preference ^d (acute)^ ↑ sucrose preference ^a,c (chronic, 10/40)^ ~ locomotor activity ^d (acute/chronic, 10/40)^	↑ hippocampal BDNF ^b (10/40)^ ~PFC BDNF ^d (acute/chronic, 10/40)^
Kodali and colleagues, 2015 [24]	Late/middle-age male Fischer 344 rats	RSV v Vehicle 4 weeks txt, 4 weeks waiting period, behavioral tests	RSV: 40, i.p.	↓ immobility time in FST ^a^	↑ BrdU+ cells ^a^ ↑ hippocampal neurogenesis ^b^ ↑ DCX newly born neurons ^c^ ↑ hippocampal microvasculature ^b^ and CA1 subfield microvasculature ^a^ ↓ astrocyte hypertrophy ^c^ ↑ hippocampal resting microglia ^a^
Li and colleagues, 2016 [25]	CORT-injected male ICR mice	CORT RSV FLX Pioglitazone × 3 weeks	CORT: 40 s.c. RSV: 50, 100, oral FLX: 20, oral pioglitazone: 10, oral	↑ sucrose preference ^b,c (50/100)^ ↓ immobility time in FST ^b,c (50/100)^ ~ locomotor activity ^d^	↓ serum CORT ^b,b (50/100)^
Liu and colleagues, 2014 [26]	CUMS-exposed male Wistar rats	Vehicle RSV (80) DES (10) CUMS + vehicle CUMS + RSV (20, 40, 80) CUMS + DES × 5 weeks	RSV: 20, 40, 80, i.p. DES: 10, i.p. Vehicle: 1% ethanol, i.p.	↑ sucrose preference ^a,a,b (20/40/80)^ ↓ immobility time in FST ^a,a (40/80)^ ↑ locomotor activity ^a (80)^	↓ serum CORT ^a (80)^ ↑ hippocampal BDNF ^b (80)^ ↑ amygdala BDNF ^b,b (40/80)^ ↑ hippocampal and amygdala pCREB/CREB ratio ^b,a (80)^ ↑ hippocampal pERK ^a,b (40/80)^ ↑ amygdala pERK ^a,a (40/80)^
iu and colleagues, 2014 [27]	CUMS-exposed male Wistar rats	Vehicle RSV CUMS + vehicle CUMS + RSV × 5 weeks	vehicle (1% ethanol) RSV: 80 i.p.	↓ escape latency in Morris water maze ^b^ ↑ exploration time in novel object recognition test ^a^	↓ serum CORT ^a^ ↑ PFC BDNF ^a^ ↑ hippocampal BDNF ^a^ ↓ hippocampal and PFC p CREB/CREB ratio ^a^ ↑ p ERK/ERK ratio ^a^
Liu and colleagues, 2016 [5]	LPS-injected adult male C57/BL6 mice	Saline + DMSO Saline + RSV LPS + DMSO LPS + RSV × 14 days	RSV: 20, i.p. LPS: 1, i.p.	↓ immobility time in FST ^b^ ↓ immobility time in TST ^a^	↓ microglia w/ activated morphologies ↓ Ib-A1 immunoreactivity ^b^ ↑ BrdU+ cells ^b^ ↑ DCX+ neurons ^b^ ↑ type-1 RGL cells ^a^ ↑ symmetric division of RGL cells ^b^ ↓ hippocampal NF-κB expression ^b^
Liu and colleagues, 2016 [28]	CUMS-exposed male Wistar rats	Vehicle RSV CUMS + vehicle CUMS + RSV CUMS + ketamine × 4 weeks	RSV: 80, i.p. Ketamine: 20, i.p. Vehicle: 1% ethanol, i.p.	↑ sucrose preference ^a^ ↓ immobility time in FST ^b^ ↑ locomotor activity ^a^	↓ MDA in hippocampus & PFC ^c^ ↑ SOD in hippocampus ^c^ & PFC ^b^ ↑ phosphorylated mTOR in hippocampus ^a^ & PFC ^a^ ↑ p-Akt in hippocampus ^a^ & PFC ^a^
López and colleagues, 2014 [29]	male CD1 mice	vehicle (saline) DMSO 1% DMSO 10% RSV OXO 4 buproprion citalopram DES imipramine moclobemide nisoxetine nomifensine	RSV: 2.5, 5, 10, i.p. OXO 4: 1, i.p. buproprion: 10 i.p. citalopram: 20 i.p. DES: 35 i.p. imipramine: 35 i.p. moclobemide: 35 i.p. nisoxetine: 2.5 i.p. nomifensine: 2.5 i.p.	↓ immobility time in FST ^a(10)^	N/A
Pang and colleagues, 2015 [30]	middle cerebral artery occluded male Sprague-Dawley rats	sham/vehicle MCAO MCAO + RES MCAO + imipramine × 7 days pre-surgery; tested either day 8 or days 20–21	RSV: 10, 20, 40, oral imipramine: 10 i.p.	↑ sucrose preference ^a,b (20/40)^ ↓ immobility time in FST ^a,c (20/40)^ ~ locomotor activity ^d^	↑ hippocampal BDNF ^a^ ↑ hippocampal β-actin ^a^ ↓ adrenal gland index ^b (40)^ ↓ CRF expression in FC, hippocampus, and hypothalamus ^b,c (20/40)^ ↓ glucocorticoid receptor expression in FC ^b,c (20/40)^, hippocampus ^b,c (20/40)^, and hypothalamus ^a (40)^ ↑ BDNF expression in FC ^a,a (20/40)^, hippocampus ^b,b (20/40)^, and hypothalamus ^b,b (20/40)^
Sakr and colleagues, 2015 [31]	CUMS- exposed male Sprague-Dawley rats	CUMS + watercontrol CUMS + FLX water CUMS + RSV CUMS + FLX/RSV × 4 weeks ctrl + water ctrl + FLX ctrl + RSV ctrl + FLX/RSV	FLX: 10, oral RSV: 20, oral	↑ sucrose preference ^a^ ↑ immobility time in FST ^a^	↓ serum CORT ^a^ ↓ 5-HT in cerebral cortex and hippocampus ^a^ ↑ serum testosterone ^a^ ↑ testicular SOD ^a^ ↑ testicular CAT ^a^ ↑ testicular GSH ^a^ ↓ testicular MDA ^a^
Wang and colleagues, 2013 [32]	male Kunming mice	Vehicle RSV FLX × 21 days; tests followed	RSV: 20, 40, 80, i.p. FLX: 10, i.p. Vehicle: 1% ethanol, i.p., 0.9% NaCl, i.p.	↓ immobility time in FST ^a,b,c (20/40/80)^ ↓ immobility time in TST ^a,c,c (20/40/80)^ ~ locomotor activity ^d^	↓ serum CORT ^b(80)^ ↑ PFC BDNF ^c,c,c (20/40/80)^ ↑ hippocampal BDNF ^c,c (40/80)^ ↑ pERK 1/2 in PFC ^a,a,c^ & hippocampus ^a,b,c (20/40/80)^
Wang and colleagues, 2016 [33]	CRS-exposed male Wistar rats	RSV FLX CRS exposed 30 min after injection × 21 days	RSV: 80, i.p. FLX: 10, i.p.	↑ sucrose preference ^a^ ↓ immobility time in FST ^a^ ~ locomotor activity ^d^	↑ hippocampal BDNF ^a^ ↑ PFC BDNF ^a^ ↑ BDNF/GFAP immunoreaction in hippocampus ^c^ ↑ pERK/ERK ratio in hippocampus ^b^ and PFC ^b^ ↑ bcl-2 hippocampal & PFC mRNA ^a^ ↓ hippocampal BAX mRNA ^b^
Wang and colleagues, 2018 [34]	ouabain- exposed female J20 mice	ouabain + PBS (control) ouabain + RSV × 10 weeks	RSV: 10, oral	↑ distance moved ^a^ ↑ path efficiency ^a^ ↓ time to recognize novel object ^a^ ↓ Rankin score ^a^	↓ plasma IL-1β, IL-17A, IL-8 and TNF-α ^a^ ↓ serum H3R ^a^ ↓ hippocampal plasma IL-1β, IL-17A, IL-8 and TNF-α ^a^ ↓ hippocampal CAT, SOD, GSH and NEG ^a^ ↓ hippocampal COX-2 expression ^a^ ↓ hippocampal neuron apoptosis ^a^ ↑ hippocampal neuron P53 and Bcl-2 ^a^ ↑ hippocampal neuron NETRIN1 and NRG3 ^a^ ↓ hippocampal neuron cAMP ^a^
Xu and colleagues, 2010 [7]	male ICR mice	RSV Moclobemide (MAOI) Imipramine (TCA) Fluoxetine (SSRI) Treated w/PCPA or vehicle prior to FST & TST; treated w/apomorphine or vehicle after RSV Txt; behavioral tests 30 min post-RSV	RSV: 20, 40, 80, i.g. Moclobemide: 20, i.p. Imipramine: 20, i.p. Fluoxetine: 10, i.p. PCPA: 300, i.p. Apomorphine: 16, s.c. Vehicle	↓ immobility time in FST ^a,b,c (20/40/80)^ ↓ immobility time in TST ^a,a,a (20/40/80)^ ~ locomotor activity ^d^	↑ 5-HT ^b (80)^, norepinephrine ^a (80)^ & dopamine ^a (80)^ in FC ↓ 5-HIAA/5-HT ratio in FC ^b (80)^ ↑ hippocampal 5-HT ^a,b (40/80)^ & norepinephrine ^a (80)^ ↓ hippocampal 5-HIAA/5-HT ratio ^a (80)^ ↓ MAO-A activity ^a,b,c (20,40,80)^ ↓ MAO-B activity ^b (80)^

^a^*p* < 0.05, ^b^ *p* < 0.01, ^c^ *p* < 0.001, ^d^ *p* > 0.05 ~ No change, * ug/kg/d (All others mg/kg/day). 5-HIAA, 5-hydroxyindoleacetic acid; 5-HT, serotonin; ATP, adenosine triphosphate; bcl-2,ind B-cell lymphoma 2; BAX, bcl-2-like protein 4; BDNF, brain-derived neurotrophic factor; BrdU, bromodeoxyuridine; CORT, corticosterone; CRH, corticotrophin-releasing hormone; CRS, chronic restraint stress; CUMS, chronic unpredictable mild stress; DES, desipramine; DCX, doublecortin; DMSO, dimethyl sulfoxide; FC, frontal cortex; FLX, fluoxetine; FST, forced swimming test; GFAP, glial fibrillary acidic protein; GSH, glutathione; GSK, glycogen synthase kinase; i.c.v., intracerebroventricular; i.g., intragastric; i.p., intraperitoneal; IL, interleukin; LPS, lipopolysaccharide; LT4, levothyroxine; MAO-A, monoamine oxidase A; MAO-B, monoamine oxidase B; MAOI, monoamine oxidase inhibitor; MCAO, middle cerebral artery occlusion; MDA, malondialdehyde; NEG, neuroglobin; mTOR, mammalian target of rapamycin; OXO 4, 5-methoxyoxoisoaporphine; pβ-catenin, phosphorylated β-catenin; PCPA, parachlorophenylalanine; pCREB, phosphorylated cAMP response element binding protein; PFC, prefrontal cortex; pGSK, phosphorylated glycogen synthase kinase; pNF-κB, phosphorylated NF-κB; RGL, radial-glia-like cell; RSV, resveratrol; s.c., subcutaneous; SCH, subclinical hypothyroidism; SOD, superoxide dismutase; SSR, selective serotonin reuptake inhibitor; TCA, tricyclic antidepressant; TNF, tumor necrosis factor; TRH, thyrotropin-releasing hormone; TSH, thyroid-stimulating hormone; TST, tail suspension test; Txt, treatment.

**Table 2:** Comparison of resveratrol with antidepressant drugs.

Author, Year	Animal Model	Txt Duration	Dosages (mg/kg/Day)	Comparative Effectiveness of RSV
Ahmed and colleagues, 2014 [17]	reserpine-injected adult male Wistar rats	3 day	RSV: 15, 30, 60, oral FLX: 24, oral	>FLX: Liver GSH ^(60)^, liver MDA ^(30/60)^ =FLX: FST ^(60)^, 5-HT ^(15/30/60)^, norepinephrine ^(60)^, dopamine ^(15/30/60)^, brain MDA ^(60)^ < FLX: OFT
Ali and colleagues, 2015 [18]	CORT-injected male Swiss albino mice	21 days	RSV: 80, oral FLX: 15, oral	=FLX: Sucrose preference, immobility time in FST, immobility time in TST, serum CORT, hippocampal BDNF
Ge and colleagues, 2013 [21]	CUMS-exposed male Sprague-Dawley rats	21 days	RSV: 15, i.p. FLX: 2, i.p.	=FLX: Sucrose preference, immobility time in FST, immobility time in TST, serum MDA, serum CORT <FLX: CRH mRNA expression
Huang and colleagues, 2013 [23]	male ICR mice	4 days	RSV: 1.25, 2.5, 10, 20, 40, 80, oral + piperine: 2.5 i.p. FLX: 10 i.p. Imipramine: 10 i.p.	=FLX: Immobility time in FST ^(10/20)^, immobility time in TST ^(10/20)^ =Imipramine: locomotor activity; reserpine-induced hypothermia ^(10,20)^ and ptosis ^(20)^; FC 5-HT ^(10,20)^, norepinephrine ^(20)^, dopamine ^(20)^, and 5-HIAA/5-HT ratio ^(20)^
Li and colleagues, 2016 [25]	CORT-injected male ICR mice	21 days	RSV: 50,100, oral FLX: 20, oral	=FLX: Sucrose preference ^(50/100)^, immobility time in FST ^(50/100)^, CORT ^(50/100)^
Liu and colleagues, 2014 [26]	CUMS-exposed male Wistar rats	5 weeks	RSV: 20, 40, 80, i.p. DES: 10, i.p.	=sucrose preference ^(20/40/80)^, immobility time in FST ^(40/80)^, crossing and grooming in OFT ^(80)^, serum CORT ^(80)^ BDNF in hippocampus ^(80)^ and amygdala ^(40/80)^, p-CREB in hippocampus ^(80)^ and amygdala ^(80)^, p-ERK in hippocampus ^(40/80)^ and amygdala ^(40/80)^
Liu and colleagues, 2016 [28]	CUMS-exposed male Wistar rats	4 weeks	RSV: 80, i.p. Ketamine: 20, i.p	=Ketamine: Sucrose preference; immobility time in FST; OFT; PFC and hippocampal MDA and SOD; phosphoylated mTOR and Akt
López and colleagues, 2014 [29]	male CD1 mice	<1 day	RSV: 2.5, 5, 10, i.p. buproprion: 10 i.p. citalopram: 20 i.p. desipramine: 35 i.p. imipramine: 35 i.p. moclobemide: 35 i.p. nisoxetine: 2.5 i.p. nomifensine: 2.5 i.p.	↓ immobility time in FST ^(10)^
Pang and colleagues, 2015 [30]	middle cerebral artery occluded male Sprague-Dawley rats	14 days	RSV: 10, 20, 40, oral Imipramine: 10 i.p.	=Imipramine: Sucrose preference ^(20/40)^; FST ^(20/40)^; CRF expression in hypothalamus ^(20/40)^, hippocampus ^(20/40)^ and FC ^(20/40)^; GR expression in hypothalamus ^(40)^, hippocampus ^(20/40)^, and FC ^(20/40)^; BDNF expression in hypothalamus ^(40)^, hippocampus ^(20/40)^ and FC ^(40)^ <Imipramine: adrenal gland index
Sakr and colleagues, 2015 [31]	CUMS-exposed male Sprague-Dawley rats	28 days	RSV: 20, oral FLX: 10, oral	>FLX: MDA, SOD, CAT, GSH <FLX: Sucrose preference, immobility time in FST, serum testosterone, serum CORT, hippocampal 5-HT
Wang and colleagues, 2013 [32]	male Kunming mice	21 days	RSV: 20, 40, 80, i.p. FLX: 10, i.p.	=FLX: Immobility time in FST ^(20/40/80)^, immobility time in TST ^(20/40/80)^, serum CORT ^(80)^, BDNF mRNA in hippocampus ^(40/80)^ and PFC ^(20/40/80)^, BDNF protein expression in hippocampus ^(20/40/80)^ and PFC ^(20/40/80)^
Wang and colleagues, 2016 [33]	CRS-exposed male Wistar rats	21 days	RSV: 80, i.p. FLX: 10, i.p.	=FLX: Sucrose preference; immobility time in FST; OFT; hippocampal and PFC BDNF mRNA and protein expression; hippocampal and PFC phosphorylated ERK mRNA and protein expression
Xu and colleagues, 2010 [7]	male ICR mice	4 days	RSV: 20, 40, 80, i.g. FLX: 10, i.p. Moclobemide: 20, i.p. Imipramine: 20, i.p.	>FLX: Dopamine in FC ^(80)^; 5-HIAA/5-HT ratio in FC ^(80)^, hippocampus ^(80)^ and hypothalamus ^(80)^; noradrenaline in FC ^(80)^ and hippocampus ^(80)^; MAO-A ^(20/40/80)^; MAO-B ^(80)^ =FLX: FST ^(20/40/80)^; TST ^(40/80)^; 5-HT in FC ^(80)^, hippocampus ^(40/80)^ and hypothalamus ^(80)^ >Imipramine: Dopamine in FC ^(80)^; 5-HIAA/5-HT ratio in FC ^(80)^, hippocampus ^(80)^ and hypothalamus ^(80)^; MAO-A ^(20/40/80)^; MAO-B ^(80)^ =Imipramine: FST ^(20/40/80)^; TST ^(40/80)^; apomorphine-induced hypothermia ^(20/40/80)^; 5-HT in FC ^(80)^, hippocampus ^(40/80)^ and hypothalamus ^(80)^; noradrenaline in FC ^(80)^ and hippocampus ^(80)^ =Moclobemide: MAO-A ^(20/40/80)^ >Moclobemide: MAO-B ^(80)^

5-HIAA, 5-hydroxyindoleacetic acid; 5-HT, serotonin; BDNF, brain-derived neurotrophic factor; CAT, catalase; CORT, corticosterone; CRF, corticotropin-releasing factor; CRH, corticotropin-releasing hormone; CRS, chronic restraint stress; CUMS, chronic unpredictable mild stress; ERK, extracellular signal-regulated kinase; FC, frontal cortex; FLX, fluoxetine; FST, forced swimming test; GR, glucocorticoid receptor; GSH, glutathione; i.g., intragastric; i.p., intraparitoneal ; MAO-A, monoamine oxidase A; MAO-B, monoamine oxidase B; MDA, malondialdehyde; Mtor, mammalian target of rapamycin; OFT, open field test; PFC, prefrontal cortex; RSV, resveratrol; SOD, superoxide dismutase; TST, tail suspension test.
